# Psychosocial and Behavioral Factors in Awake Bruxism—Self-Report versus Ecological Momentary Assessment

**DOI:** 10.3390/jcm10194447

**Published:** 2021-09-27

**Authors:** Alona Emodi-Perlman, Daniele Manfredini, Tamar Shalev, Alessandro Bracci, Pessia Frideman-Rubin, Ilana Eli

**Affiliations:** 1 The Maurice and Gabriela Goldschleger School of Dental Medicine, Tel-Aviv University, Tel-Aviv 6139001, Israel; tamarashalev@gmail.com (T.S.); pessia80@gmail.com (P.F.-R.); elilana@tauex.tau.ac.il (I.E.); 2 School of Dentistry, Department of Biomedical Technologies, University of Siena, 53100 Siena, Italy; daniele.manfredini75@gmail.com; 3 School of Dentistry, University of Padova, 35122 Padova, Italy; info@alessandrobracci.com

**Keywords:** awake bruxism, self-report, ecological momentary assessment, oral behaviors, psychosocial factors

## Abstract

The issue of psychosocial factors and concurrent conditions associated with AB is a relatively new approach in the study of Awake Bruxism (AB). In the present study a population of 84 dental students were assessed for probable AB with two modes of AB assessment: Single point self-report (SR) and ecological momentary assessment through a designated smartphone application (BA). The two assessment modes were compared with regard to their ability to phenotype subjects as far as the following psychosocial and behavioral variables are concerned: Gender; depression; somatization; oral behaviors; chronic pain and associated pain symptoms in the head, neck and scapula. Two-way ANOVA showed main effect of SR for the following variables: Chronic Pain Intensity score (F_(1,49)_ = 6.441, *p* < 0.02), migraine/headache (F_(1,81)_ = 7.396, *p* < 0.01), pain in neck (F_(1,81)_ = 6.726, *p* < 0.05), pain in scapula (F_(1,81)_ = 8.546, *p* < 0.005) and the oral behaviors of pushing the tongue forcefully against the teeth (F_(1,81)_ = 5.222, *p* < 0.05) and inserting the tongue between the upper and lower teeth (F_(1,81)_ = 5.344, *p* < 0.03). The effect of SR on the habit of chewing gum was borderline (F_(1,81)_ = 3.369, *p* = 0.07). Main effect of BA was found for depression (F_(1,81)_ = 6.049, *p* < 0.05), while the effect of BA on somatization was borderline (F_(1,81)_ = 3.657, *p* = 0.059). An interaction between SR and BA groups could be observed for the behavior of biting, chewing or playing with the tongue, cheeks or lips (F_(1,81)_ = 4.117, *p* < 0.05). The results suggest that a combination of a single-point self-report referring to the past 30 days, and an ecological momentary assessment supplying information about the actual timing of the report, can help us to better assess AB, as well as increase our ability to define the phenotype of subjects with AB as far as psychosocial and behavioral factors are concerned.

## 1. Introduction

Awake bruxism (AB) is defined as a masticatory muscle activity during wakefulness, which is characterized by repetitive sustained tooth contact and/or bracing or thrusting of the mandible. AB is not considered a movement disorder in otherwise healthy individuals [1].

Recently, the first steps towards the standardization of bruxism assessment were published [2]. The general structure is planned to rely on two axes: (i) Evaluation axis A—dealing with assessment of bruxism (self-reports, clinical evaluation, instrumental assessment); and (ii) etiological/risk factors axis B—dealing with etiology, risk factors and concurrent conditions associated with AB. 

The issue of psychosocial factors and concurrent conditions associated with AB will undoubtedly be a major factor in future AB studies. Barbosa Camara-Souza et al. [3] presented significant correlations between AB and anxiety, depression, stress, oral health related quality of life and certain behaviors during wakefulness. 

Ecological momentary assessment (EMA) is a new semi-instrumental approach for assessing AB, which is intended to decrease the problematic issue of retrospective recall. EMA refers to real-time reporting of a certain behavior, feeling or condition [4]. Smartphone application(s) provide an easy way to employ EMA through alerts sent to the user, in order to complete the relevant assessment mode [5]. EMA reduces the problem recall bias, and is an efficient way to collect data. It may also reduce the number of participants needed for the study. Nevertheless, EMA can also be frustrating for participants and requires specific skills for data analysis [6]. Emodi-Perlman et al. [7] showed that a combination between single-point self-report and EMA can promote our ability to assess AB.

The aim of the present study was to compare the ability of single-point self-report and EMA to phenotype subjects regarding the following psychosocial and behavioral variables: Gender, depression, somatization, oral behaviors, chronic pain and associated pain symptoms in the head, neck and scapula.

## 2. Materials and Methods

Detailed information about the study population and AB assessment has been previously reported [7]. 

Briefly, two hundred and thirty-eight dental students, studying at the Maurice and Gabriela Goldschleger School of Dental Medicine, Tel Aviv University, were approached in January 2020. 

Inclusion criteria included studying towards a DMD degree (6 year curriculum) and being in generally a good health. By that point, all students had received lectures about bruxism and were familiar with the syndrome. Additionally, participants were requested to participate in a dedicated meeting in which information about EMA and the aims the study were supplied. All participants had a personal smartphone. 

Exclusion criteria were as follows: 

1. Use of medications, such as SSRIs (selective serotonin reuptake inhibitors) and/or dopamine antagonists; 

2. Confirmed conditions of depression, Parkinson, gastro-intestinal reflux, peptic ulcer, bulimia, anorexia, fibromyalgia, tinnitus, oromandibular dystonia, Huntington’s disease, Tourette syndrome, hemifacial spasms, tardive dyskinesia, REM-behavior disorder, Rheumatoid arthritis, Lupus and other arthritic disease;

3. Trauma in head or jaw during past month;

4. Car accident with whiplash injury during past month.

Participants were requested to use an EMA application for at least seven consecutive days and respond to self-report questionnaires. Response rate was 62%. 

### 2.1. Information Included in the Study

1. Demographics: Age, gender;

2. AB assessment, carried out through two modes;

a. Single-point observation self-report (**SR**): 

Participants were requested to respond to questions related to their awareness to grinding, clenching, holding teeth together and/or tightening the masticatory muscles during the day. The report referred to the previous 30 days of the study. 

The scoring possibilities for each of the questions was on a scale of 0 (never) to 4 (all of the time), as proposed by the Diagnostic Criteria for Temporomandibular Disorders (DC/TMD) oral behavior checklist [8]. A score of 2, 3 or 4 on one or more of the three questions was considered as positive AB.

b. EMA with the use of a designated smartphone application (BruxApp—**BA**), as described by Bracci et al. [9]. The application (BruxApp, Pontendra, Italy) sent each participant 20 alert sounds daily over a seven-day period. After the participants received an alert, they were asked to describe the present condition of her/his teeth and jaw position according to the following parameters: Relaxed jaw muscles (**BA-relaxed**), muscle bracing (without tooth contact, **BA-bracing**), teeth contact (**BA-teeth contact**), teeth clenching (**BA-clenching**) and teeth grinding (**BA-grinding**). A minimum of 12 responded alerts per day were required to be included in the analysis [10]. Subjects who failed to complete seven days of at least 12 responses per day were excluded from the study.

3. Oral behaviors:

Oral behaviors were evaluated through questions derived from the self-report Oral Behavior Checklist [8,11]. The list refers to 21 waking-state oral behaviors. From the original list, six behaviors with a possible effect on the oro-facial musculature were chosen for the present study, according to the following oral behaviors:

- Pushing the tongue forcefully against the teeth;
- Inserting the tongue between the upper and lower teeth;
- Biting, chewing, or playing with the tongue, cheeks or lips;
- Holding objects between teeth or biting objects such as hair, pipe, pencils, pens and fingers;
- Chewing gum;
- Playing music instruments which require the use of the mouth or the head (e.g., trumpet, violin).

Participants reported the daily frequency for each oral behavior on a scale of 0-4 ranging from “none of the time” to “all of the time”. 

4. Graded chronic pain scale (GCPS) [12,13].

The GCPS is a multidimensional measure that assesses two dimensions of overall chronic pain severity: Pain intensity and pain-related disability. 

The GCPS is a valid and reliable tool, which allows grading of the global severity of chronic pain and the impact of pain on daily, social and work activities.

Subjects were requested to respond to the following items regarding oro-facial pain:

- Magnitude of present pain (VAS scale, ranging from 0—no pain to 10—unbearable pain);
- Magnitude of worst pain during past 6 months (scale as above);
- Magnitude of average pain during past 6 months (scale as above);
- Degree of interference in daily activities caused by the oro-facial pain during past 6 months (VAS scale, ranging from 0—no interference to 10—can’t perform activity;
- Degree of interference in social activities with family and friends, caused by the oro-facial pain (scale as above);
- Degree of interference in ability to work (including housework) caused by the pain (scale as above);
- Number of absent days from work/studies/housework during past 6 months due to the oro-facial pain.

The following GCPS subscales [14] were used in the present study: 

(i) Characteristic pain intensity score (**CPI**), calculated as the mean intensity ratings for reported current, worst, and average pain;

(ii) Interference score (**IS**), calculated as the mean rating for difficulty performing daily, social, and work activities.

5. Associated pain symptoms in the head, neck and scapula:

- Experiencing migraine or headache, during past 30 days;
- Experiencing pain in the neck, during past 30 days;
- Experiencing pain in the area of the scapula, during past 30 days;

Each of the above conditions was graded a scale of 0-4 ranging from “never” to “all of the time”. 

6. Depression: 

Depression was evaluated through the SCL-90 depression scale. The SCL-90 questionnaire was developed by Derrogatis et al. [15] as a general measure of psychiatric outpatient symptomatology in both clinical and research studies [16]. It has been used in numerous studies worldwide. The SCL-90 depression scale was included in the Research Diagnostic Criteria for Temporomandibular Disorders (RDC/TMD) [17]. 

The scale is comprised of 13 items, each of them scored on a 0-5 scale, ranging from 0- “not at all” to 5- “extremely”. 

7. Somatization

Somatization was evaluated through the SCL-90 somatization scale [15,17,18] as explained above. The somatization scale was included in the Research Diagnostic Criteria for Temporomandibular Disorders (RDC/TMD) [17]. It is comprised of 12 items, scoring as per the details above.

The study was approved by the ethical committee of Tel-Aviv University, approval no. 000693-1. Written informed consent was obtained from all the participants.

### 2.2. Statistics

The frequency (percentage) of the BA-relaxed condition was calculated as a percentage with respect to the answered alerts for all individuals. The frequency was calculated daily on an individual basis and individual frequency was used to calculate an average for the study population, on a daily basis [9].

Pearson correlation coefficient was used to correlate the BA-relaxed condition, as a continuous variable, with the study variables (gender, oral behaviors, CPI, IS, migraine, pain in neck/scapula, depression and somatization).

Two-way analysis of variance (ANOVA) was carried out to test between-subjects effects of the SR (positive versus negative) and the BA groups (as defined in the Results section below) and possible interactions among them, with regard to the study variables.

The level of significance was set as 0.05.

### 2.3. Results

From the 147 dental students who consented to participate in the study, 106 completed of a full BA application response (7 days/minimum, 12 responses per day). A total of 21 additional subjects were excluded due to medical conditions, as defined in the exclusion criteria. The final number of participants included in the analyses was 85. 

The mean age of the study population was 24.4 ± 2.99 years. 

### 2.4. Correlation Analyses

Pearson correlation coefficient: Previous publication [7] showed that the BA-relaxed condition was the one with the most prominent frequency, as compared to the other BA conditions. The frequency of the BA-relaxed ranged 55.5–63.7% over the 7 days of the study, with the lowest frequency recorded during the first day of report. The increase in the BA-relaxed frequency over time occurred most probably due to an increase in subjects’ self-awareness, which may have acted as a biofeedback. 

Therefore, the BA-relaxed data collected on the first day of the report were used in Pearson correlation analyses with gender, oral behaviors, CPI, IS, migraine/headache, pain in neck/scapula, depression and somatization. 

The only significant result was a negative correlation between BA-relaxed (on the first day) and Somatization (r = –0.250, *p* < 0.05).

### 2.5. Analysis of Variance (ANOVA)

a. Definition of Self-report groups (SR): Subjects were divided as AB positive and AB negative according to the single point observation self-report (SR). Groups were defined as **SR-positive** (N = 55; 64.7%) versus **SR-negative** (N = 30; 35.3%) as described in Materials and Methods. The SR-positive and SR-negative groups were compared with regard to the study variables (gender, oral behaviors, CPI, IS, migraine, pain in neck/scapula, depression and somatization).

b. Definition of EMA assessment groups (BA): The prevalence of the BA-relaxed condition (around 60%, as reported by Emodi-Perlman et al.) [7], coincided with the study of Zani et al. [19], who reported an average frequency of 62% for the BA-relaxed condition. 

Therefore, BA groups in the present study were defined according to their report of the BA-relaxed condition as follows: (i) Subjects whose report of the relaxed condition was ≥60% (N = 40; 47%) were referred to as BA-negative Awake Bruxism **(BA-nAB**) versus (ii) subjects whose report of the relaxed condition was < 60% (N = 45; 53%) were referred to as BA-positive Awake Bruxism (**BA-pAB**), and compared as detailed above. 

Two-way ANOVA was used to analyze the difference between the means of the four groups (SR-positive, SR-negative, BA-nAB, BA-pAB), in order to learn how the two independent variables (SR, BA) in combination, affect the dependent variables of gender, oral behaviors, CPI, IS, migraine/headache, pain in neck/scapula, depression and somatization. The analyses tested, at the same time, the following alternative hypotheses regarding each of the above-mentioned dependent variables: (i) There is a difference in the group means of the SR variable (main effect); (ii) there is a difference in the group means of the BA variable (main effect); and (iii) there is an interaction effect between one independent variable (SR) and the other independent variable (BA).

Distribution of participants among the four groups is presented in Table 1. 

Main effect of SR (SR positive vs. SR negative) was found for the following variables: CPI (F_(1,49)_ = 6.441, *p* < 0.02), migraine/headache (F_(1,81)_ = 7.396, *p* < 0.01), pain in neck (F_(1,81)_ = 6.726, *p* < 0.05), pain in scapula (F_(1,81)_ = 8.546, *p* < 0.005) and the oral behaviors of pushing the tongue forcefully against the teeth (F_(1,81)_ = 5.222, *p* < 0.05) and inserting the tongue between the upper and lower teeth (F_(1,81)_ = 5.344, *p* < 0.03). The effect of SR on the habit of chewing gum was borderline (F_(1,81)_ = 3.369, *p* = 0.07) (Table 2). 

**Figure 1:**
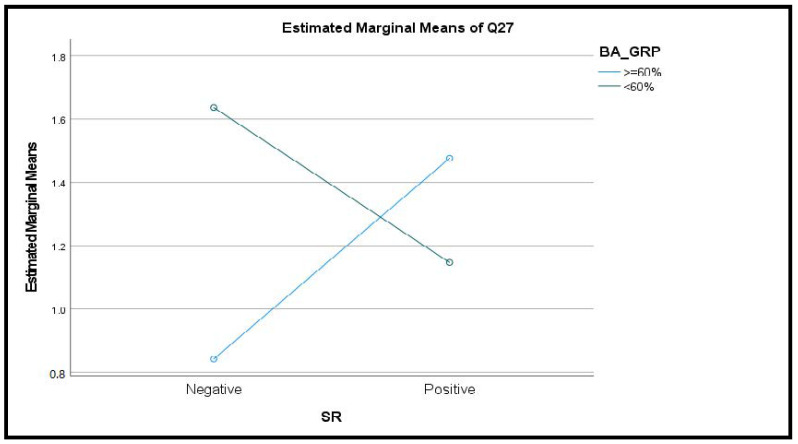
Interaction between SR and BA groups with regard to biting, chewing or playing with the tongue, cheeks or lips.

An interaction between SR and BA groups was observed for the behavior of biting, chewing or playing with the tongue, cheeks or lips (F_(1,81)_ = 4.117, *p* < 0.05, Figure 1). 

## 3. Discussion

The etiology of bruxism combines genetic factors with various psychosocial aspects such as stress sensitivity, anxiety, and poor coping skills [20,21,22,23,24].

The assessment accuracy of single point self-reported bruxism is considered to be low [3,25,26,27]. The semi-instrumental EMA approach via a smartphone app (BA) is a relatively new strategy to assess AB. Its validity and reliability still need to be determined. Emodi-Perlman et al. [7] suggested that a combination between the two modes has the potential to more effectively assess AB. 

A previous study [7] showed that the most prevalent condition recorded by the BA application among healthy subjects was BA-relaxed and suggested to minimize the reported conditions to BA-relaxed versus one or two other conditions describing muscular strain. Previous publications have been recommended to assess bruxism activity in its continuum [1,19,28], rather than to determine the accepted cut-off points. However, an initial attempt to use the BA-relaxed condition as a continuous variable in correlation with other psychosocial and behavioral variables yielded almost no results.

Several studies showed that the frequency of the BA-relaxed condition in healthy young adults, is around 60% [7,9,19]. Therefore, we chose the 60% point as a way to distinguish between subjects whose masticatory muscles are mostly relaxed, who most probably do not suffer from AB (BA-nAB, ≥60%), those who tend to be engaged in various muscular activities (such as bringing their teeth in contact, grinding, clenching or bracing), and most probably do present some degree of AB (BA-pAB, <60%). 

Initial comparisons within the SR (negative vs. positive) and the BA (BA-nAB vs. BA-pAB) groups showed that the two assessment modes behave differently with regard to the psychosocial and behavioral variables examined in the present study. 

There were no differences in age and gender among the four group combinations (BA-nAB/ SR negative; BA-nAB/ SR positive; BA-pAB/ SR negative; BA-pAB/ SR positive). The two groups which presented seemingly “consistent” reports indicating either an absence of AB (BA-nAB/SR negative) or a presence of AB (BA-pAB/SR positive), accounted for the majority of subjects (63%), supporting the notion that a combination between SR and BA may promote our ability to assess AB [7]. 

The findings that SR-positive and SR-negative subjects differed in relation to oral behaviors such as pushing the tongue against teeth and inserting tongue between upper and lower teeth is not surprising as these oral behaviors resemble, or may be part, of AB. The differences between these two groups as far as pain in the head and neck area (pain in neck or scapula, migraine/headache, CPI) coincides with previous publications which show that bruxism (sleep or awake) is often associated with tension headache and migraine [29]. 

In their comprehensive review, Baad-Hansen et al. [30] showed that in adults AB may be positively associated with pain located in the masticatory muscles. This association was supported both by studies using self-report, as well as in an instrumental diagnosis of awake bruxism. The authors indicated that studies based on self-reports are more indicative of positive associations between bruxism and pain than studies employing clinical examinations and instrumental evaluations of muscle activity. 

In the present findings no differences were found between the BA groups (BA-nAB vs. BA-pAB) with regard to possibly associated pain symptoms (CPI, headache/migraine, pain in neck/scapula). The reason may lie in the fact that BA refer to the very moment of response, while the single point self-report (SR) and reports of possibly associated pain symptoms refer to a recall of the past 30 days. 

While the BA groups did not differ from one another regarding oral behaviors and associated pain symptoms differences between the two groups emerged with regard to depression and somatization. This confirms the findings of Barbosa Câmara-Souza et al. [3] who showed that anxiety, depression and stress correlate with AB as assessed by the BA application. The interaction between SR and BA groups regarding the behavior of biting, chewing or playing with the tongue, cheeks or lips, which shows that SR and BA behave in an opposite way with regard to this variable, is intriguing and calls for further evaluation. 

The present study was based on a relatively homogenous population of young healthy adults (dental students). Still, a relatively high percentage of subjects were assessed as AB positive according to self-report (SR-positive, 64.7%) or as belonging to the BA-pAB group (53%). This may be due to stress that many dental students experience during their studies [31,32]. Soto-Goni et al. [33] showed that subjects with awake bruxism show larger levels of anxiety, somatization, and neuroticism but also display more adapted coping strategies than TMD patients. The authors suggested that AB may play a positive role in stress coping, which would be compatible with the hypothesis of mastication as a means of relieving psychological tension. This may possibly be the case in the present population. 

## 4. Conclusions

Psychosocial and behavioral phenotyping of subjects with AB is a complex issue. Present results strengthen the suggestion to include two axes in bruxism assessment: one dealing with self-reports, clinical evaluations etc., and another referring to etiology, risk factors and concurrent conditions associated with the condition [2]. A combination between single point self-report (which refers to the past 30 days) and an ecological momentary assessment (which supplies information about the actual moment of the self-report) can increase our understanding of AB. It can help us better assess AB, and can also increase our ability to define the phenotype of subjects with AB.

## 5. Study Limitations

The main obstacle in AB studies lies in the difficulty assessing AB in large populations in an accessible and reliable way. While both self-report and clinical assessment present some degrees of diagnostic sensitivity, the gold standard is still electromyography (EMG) recordings, a process that might be problematic for the continuous evaluation of subjects’ muscular activity during daytime. 

In the present study, AB was assessed using a single-point self-report and EMA. AB was not objectively confirmed through an instrumental tool as EMG. The psychosocial and behavioral aspects studied were limited, and the associated pain syndromes addressed only few limited conditions. Further studies should be carried out to increase our understanding of the AB phenomenon. These studies should be performed with wider and more diverse populations, with reference to additional psychosocial aspects (e.g., anxiety), and possibly associated painful conditions (e.g., TMD) and additional oral behaviors (e.g., the entire OBC list). 

## Figures and Tables

**Table 1:** Subjects’ distribution according to SR and BA groups.

	BA	SR-Negative	SR-Positive	Total
SR *				
BA-nAB		19 (22.4%) **	21 (24.7%)	40 (49.1%)
BA-pAB		11 (12.9%)	34 (40.6%) **	45 (53.5%)
Total		30 (35.5%)	55 (65.3%)	85 (100%)

* Number (% in parenthesis); ** Groups presenting seemingly “consistent” reports of either an absence of AB (BA-nAB/SR negative) or a presence of AB (BA-pAB/SR positive). There were no differences among groups with regard to age or gender. Neither the main effects of the SR and/or the BA groups, nor interactions between groups, were found regarding to the IS score or the oral habit of holding objects between teeth/ biting objects.

**Table 2:** Significant differences between the SR groups (2-way ANOVA).

Variable	SR-Positive *	SR-Negative
CPI	29.41 (19.85)	13.84 (18.35)
Migraine/headache	1.16 (0.78)	0.60 (0.72)
Pain in neck	0.85 (0.99)	0.37 (0.71)
Pain in scapula	1.16 (1.08)	0.50 (0.82)
Pushing tongue against teeth	1.22 (1.34)	0.47 (0.97)
Inserting tongue between teeth	0.71 (1.10)	0.23 (0.43)
Chewing Gum	1.62 (1.08)	1.20 (0.71)

* Mean score (SD in parenthesis), Main effect of BA groups (BA-nAB vs. BA-pAB) was found for depression (mean of 4.27 ± 5.89 in the BA-nAB group versus 9.91 ± 11.54 in the BA-pAB group; F_(1,81)_ = 6.049, *p* < 0.05). The effect of BA on somatization was borderline (mean of 1.60±2.27 in the BA-nAB group versus 3.22 ± 3.46 in the BA-pAB group; F_(1,81)_ = 3.657, *p* = 0.059).

## Data Availability

Additional data are available upon request from the first author (A.E.-P.).
